# Cardiac fibroblast-specific p38α MAP kinase promotes cardiac hypertrophy *via* a putative paracrine interleukin-6 signaling mechanism

**DOI:** 10.1096/fj.201701455RR

**Published:** 2018-03-30

**Authors:** Sumia A. Bageghni, Karen E. Hemmings, Ngonidzashe Zava, Christopher P. Denton, Karen E. Porter, Justin F. X. Ainscough, Mark J. Drinkhill, Neil A. Turner

**Affiliations:** * Leeds Institute of Cardiovascular and Metabolic Medicine, School of Medicine, University of Leeds, Leeds, United Kingdom; and; † Centre for Rheumatology, Division of Medicine, University College London, London, United Kingdom

**Keywords:** heart, cardiomyocyte, microRNA, isoproterenol, mouse

## Abstract

Recent studies suggest that cardiac fibroblast-specific p38α MAPK contributes to the development of cardiac hypertrophy, but the underlying mechanism is unknown. Our study used a novel fibroblast-specific, tamoxifen-inducible p38α knockout (KO) mouse line to characterize the role of fibroblast p38α in modulating cardiac hypertrophy, and we elucidated the mechanism. Myocardial injury was induced in tamoxifen-treated Cre-positive p38α KO mice or control littermates *via* chronic infusion of the β-adrenergic receptor agonist isoproterenol. Cardiac function was assessed by pressure–volume conductance catheter analysis and was evaluated for cardiac hypertrophy at tissue, cellular, and molecular levels. Isoproterenol infusion in control mice promoted overt cardiac hypertrophy and dysfunction (reduced ejection fraction, increased end systolic volume, increased cardiac weight index, increased cardiomyocyte area, increased fibrosis, and up-regulation of myocyte fetal genes and hypertrophy-associated microRNAs). Fibroblast-specific p38α KO mice exhibited marked protection against myocardial injury, with isoproterenol-induced alterations in cardiac function, histology, and molecular markers all being attenuated. *In vitro* mechanistic studies determined that cardiac fibroblasts responded to damaged myocardium by secreting several paracrine factors known to induce cardiomyocyte hypertrophy, including IL-6, whose secretion was dependent upon p38α activity. In conclusion, cardiac fibroblast p38α contributes to cardiomyocyte hypertrophy and cardiac dysfunction, potentially *via* a mechanism involving paracrine fibroblast-to-myocyte IL-6 signaling.—Bageghni, S. A., Hemmings, K. E., Zava, N., Denton, C. P., Porter, K. E., Ainscough, J. F. X., Drinkhill, M. J., Turner, N. A. Cardiac fibroblast-specific p38α MAP kinase promotes cardiac hypertrophy *via* a putative paracrine interleukin-6 signaling mechanism.

The p38 family of stress-activated MAPKs has an important role in cardiac signaling and is activated in both acute and chronic cardiac pathologies including myocardial infarction, left ventricular (LV) remodeling, contractile dysfunction, arrhythmia, and heart failure (1–4). A host of preclinical studies have demonstrated that p38 MAPK inhibition can reduce the adverse consequences of cardiac injury or stress (1–4).

There are 4 known p38 subtypes (α, β, γ, and δ); p38α and p38β share sequence homology and, unlike p38γ and p38δ, are inhibited by the pyridinyl imidazole class of p38 inhibitors (*e.g.*, SB203580). The expression and function of individual p38 subtypes varies in a cell- and tissue-dependent manner; p38α is the most highly expressed subtype in the heart, with lower levels of p38γ and p38δ, and little or no expression of p38β (5, 6). p38α knockout (KO) mice are not viable because of an essential role for this subtype in placental development (7).

Most studies on p38 subtypes in the heart have focused on the α and β subtypes (1–4). *In vitro* experiments involving ectopic overexpression of p38α/β suggested a role for the β rather than the α subtype in stimulating cardiomyocyte hypertrophy (8, 9). However, the relatively low endogenous expression of p38β in heart tissue, compared with p38α and other p38 subtypes (5, 6), may question the physiologic importance of those findings. Several studies in a variety of species and cardiac-injury models have reported that combined pharmacologic inhibition of p38α and β is effective at reducing cardiac hypertrophy (10). Similar cardioprotective effects were obtained with a p38α-selective inhibitor in an isoproterenol (ISO)-induced model of cardiac hypertrophy and dysfunction in rats (11), which suggested it was p38α, rather than p38β, which is important in mediating cardiac hypertrophy *in vivo*. However, when p38α has been selectively inhibited in cardiomyocytes *in vivo* (with either cardiomyocyte-specific KO or dominant-negative approaches), there was no apparent benefit on cardiac or cardiomyocyte hypertrophy, and some studies reported worsened hypertrophy (12–15). Similar negative results were obtained with cardiomyocyte-restricted expression of dominant-negative p38β (13). Thus, there is clear discord between pharmacologic and cardiomyocyte-specific targeted genetic approaches in ascribing roles to p38α and p38β in cardiac hypertrophy.

A possible unifying explanation for these disparate *in vivo* data relates to cell type specificity. Although pharmacologic inhibitors and global KO models affect all cell types, cardiac-specific α-myosin heavy chain (α-MHC)-driven genetic models (KO or dominant negative) specifically target cardiomyocytes. It has been widely assumed that the inhibitory effects of p38 inhibitors on cardiac hypertrophy are due to direct inhibition of p38 activity in cardiomyocytes, with little attention given to other cardiac cell types.

Cardiac fibroblasts are one of the most-prevalent, nonmyocyte cell populations in the heart, accounting for between one-fifth and two-thirds of cardiac cells, depending on the species (16–18). Although traditionally viewed solely in relation to extracellular matrix remodeling, cardiac fibroblasts are now acknowledged as being important nodal regulators of multiple aspects of cardiac function under both physiologic and pathophysiologic conditions (19–21). Importantly, recent studies have revealed that fibroblasts have a critical role in inducing cardiomyocyte hypertrophy *in vivo* through paracrine secretion of growth factors and other signaling molecules (22, 23).

We have previously shown (24) that p38α is the major subtype expressed by human cardiac fibroblasts, and that it is important for secretion of several bioactive molecules, including matrix metalloproteinase-3 and IL-6 (24–26). Cardiac-fibroblast p38α has recently been reported to have an important role in myocardial fibrosis after ischemic injury or chronic neurohumoral stimulation by driving myofibroblast differentiation and profibrotic signaling (27). In addition, fibroblast-specific KO of p38α reduced cardiac hypertrophy (ventricular to body weight ratio) induced by chronic neurohumoral stimulation (27). It has been postulated that fibroblast p38α may, therefore, be important for regulating fibroblast-to-myocyte crosstalk, resulting in cardiomyocyte hypertrophy (28).

To address the specific role of fibroblast p38α in modulating cardiac hypertrophy *in vivo*, we developed an inducible, fibroblast-targeted p38α KO mouse model and investigated cardiac function and cellular and molecular changes in a model of cardiac hypertrophy induced by chronic β-adrenergic stimulation. Our data provide evidence that cardiac fibroblast p38α is integral to ISO-induced cardiac hypertrophy and dysfunction and that this may be due to a paracrine signaling mechanism involving fibroblast secretion of the cardiomyocyte hypertrophy-inducing factor, IL-6.

## MATERIALS AND METHODS

### Animal welfare

All animal procedures were performed in accordance with the Animal Scientific Procedures Act of 1986 under UK Home Office authorization after review by the University of Leeds Animal Welfare and Ethical Review Committee. Mice were maintained in individually ventilated cages at 21°C, 50–70% humidity, 12-h light/dark cycle with Pure-o’Cel paper bedding (Datesand Group, Manchester, United Kingdom) and *ad libitum* access to water and RM1 Diet (Special Diets Services, Witham, United Kingdom).

### Generation of KO mouse models

**Figure 1:**
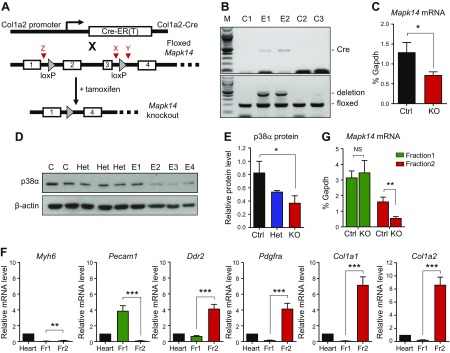
Inducible fibroblast-specific deletion of p38α in mouse heart. *A*) Schematic diagram of the deletion strategy, combining Col1a2-Cre-ER(T) mice with floxed *Mapk14* mice. Deletion of exons 2–3 occurs after tamoxifen injection, which activates Cre-ER(T). Red arrowheads denote position of genotyping primers X, Y, and Z (see Materials and Methods). *B*) Genotyping PCR showing effective exon 2/3 deletion in ear notch samples from tamoxifen-injected, experimental Cre-positive Mapk14^fl/fl^ mice (E1–2) compared with control Cre-negative Mapk14^fl/fl^ mice (C1–3). Top: Cre primers (Cre = 408 bp). Bottom: *Mapk14* exon 2/3 floxed/deletion primers. Deletion (Z + Y) = 411 bp; floxed (X + Y) = 188 bp; and M = 100-bp ladder. *C*) Real-time RT-PCR analysis of *Mapk14* mRNA levels in primary cultures of cardiac fibroblasts from control (Ctrl) Cre-negative *Mapk14*^fl/fl^ mice (*n* = 6) and Cre-positive *Mapk14*^fl/fl^ KO mice (*n* = 8) after tamoxifen injection. *P < 0.05. *D*) Immunoblot analysis of p38α protein in primary cultures of cardiac fibroblasts from control Cre-positive *Mapk14*^wt/wt^ (C), heterozygous Cre-positive *Mapk14*^wt/fl^ (Het), and experimental Cre-positive *Mapk14*^fl/fl^ mice (E1–4) after tamoxifen injection. β-actin loading control. *E*) Densitometric analysis of p38α protein expression relative to β-actin in control, heterozygous (Het), and experimental (KO) cells. *P < 0.05. *F*) Characterization of nonmyocyte, isolated cell fractions from collagenase-digested mouse hearts (*n* = 7). Fr1, endothelial cells and leukocytes; Fr2, cardiac fibroblasts. Bar charts show quantitative RT-PCR data for mRNA levels of cell type–specific marker genes. Cardiomyocyte marker, *Myh6*; endothelial marker, *Pecam1*; fibroblast markers, *Ddr2*, *Pdgfra*, *Col1a1*, and *Col1a2*. All data normalized to *Gapdh* mRNA levels and expressed relative to whole heart. **P < 0.01, ***P < 0.001. *G*) Real-time RT-PCR analysis of *Mapk14* mRNA levels in isolated cell fractions from hearts of control Cre-negative *Mapk14*^fl/fl^ mice (*n* = 3) and experimental Cre-positive *Mapk14*^fl/fl^ KO mice (*n* = 4) after tamoxifen injection. NS, not significant. **P < 0.01.

A tamoxifen-inducible fibroblast-specific p38α KO (Fb-p38α KO) mouse line was established by crossing C57BL/6 mice expressing fibroblast-specific, tamoxifen-inducible Cre recombinase [Col1a2-Cre-ER(T)] (29, 30), with C57BL/6 mice expressing a modified p38α (*Mapk14*) gene with exons 2–3 flanked by loxP sites (31) (**Fig. 1*A*** and Supplemental Fig. 1). The Col1a2-Cre-ER(T) line was obtained from Chris Denton (University College London) (29, 30), and the *Mapk14*^fl/fl^ mice were obtained from Juan-Jose Ventura (University of Cambridge, Cambridge, United Kingdom). The Col1a2-Cre-ER(T) line induces gene deletion in cardiac (and other) fibroblasts without effects on cardiomyocytes, endothelial cells, smooth muscle cells, progenitor cells, pericytes. or macrophages (32, 33). Fb-p38α KO mice (*i.e.*, Cre-positive *Mapk14*^fl/fl^) were compared alongside control littermates (*i.e.*, Cre-negative *Mapk14*^fl/fl^) for the main experimental protocols. Mice were injected with tamoxifen dissolved in corn oil (100 mg/kg/d, i.p. for a consecutive 5 d) at 3 wk old to induce Cre activity and facilitate loxP-directed deletion.

The global IL-1 receptor (*Il1r1*) KO mouse line was generated by crossing PGK-Cre global deleter mice (The Jackson Laboratory, Bar Harbor, ME, USA) with floxed *Il1r1* mice (34) (provided by E. Pinteaux, University of Manchester, Manchester, United Kingdom).

### Genotyping PCR

DNA was extracted from ear notch samples with the phenol:chloroform extraction method after incubation of samples overnight at 37°C in lysis buffer [50 mM EDTA, 10 mM Tris-HCl (pH 8.0), 1% (w/v) SDS, 0.5 mg/ml proteinase K]. End-point PCR (94°C for 3 min; 35 cycles of 94°C for 30 s, 62°C for 30 s, and 72°C for 30 s; 72°C 3 for min, and a 4°C hold) was performed with specific primer pairs: Cre forward 5′-GCATTACCGGTCGATGCAACGAGTGATGAG-3′; Cre reverse 5′-GAGTGAACGAACCTGGTCGAAATCAGTGCG-3′; p38fl forward (X) 5′-CTACAGAATGCACCTCGGATG-3′; p38fl reverse (Y) 5′-AGAAGGCTGGATTTGCACAAG-3′; and p38 deletion forward (Z); 5′-CCAGCACTTGGAAGGCTATTC-3′; p38 deletion reverse (Y) 5′-AGAAGGCTGGATTTGCACAAG-3′. Agarose gel electrophoresis was used to identify the presence of Cre (408 bp), floxed *Mapk14* (188 bp floxed, 121 bp wild type), and the deletion of *Mapk14* exons 2-3 (411 bp).

### Cardiac fibroblast culture

Cardiac fibroblasts were cultured from mouse hearts by collagenase digestion, as previously described (35). Briefly, hearts were thoroughly washed in PBS and minced before digestion with type II collagenase (2 mg/ml, 600 IU/ml; Worthington Biochemical, Lakewood, NJ, USA) at 37°C for 90 min with regular shaking. Cells were pelleted by centrifugation and washed twice in culture medium before seeding into 3 wells of a 6-well tissue culture plate (one each for DNA, RNA, and protein analysis) or a 25-cm^2^ cell culture flask for subsequent passaging. Nonadherent cells were removed after 30 min, and cells incubated with full growth medium (DMEM + 10% fetal calf serum). Cells were washed twice with PBS the next day to remove residual nonadherent cells, and fresh growth medium was added. Primary cultures of cells were analyzed for p38α expression 4 d after plating. Passage 1 cells were used for *in vitro* mechanistic studies.

### Cardiac cell fractionation

Collagenase-digested hearts were filtered through a 30-μm magnetic antibody cell separation (MACS) SmartStrainer (Miltenyi Biotec, Bergisch Gladbach, Germany) to remove cardiomyocytes. Nonmyocytes were separated into 2 fractions: nonfibroblasts (endothelial cells and leukocytes) and fibroblasts with a Cardiac Fibroblast MACS Separation Kit (Miltenyi Biotec). RNA was extracted from cell fractions, and quantitative RT-PCR was used to quantify mRNA for cell type–specific marker genes and *Mapk14*. Gene expression levels were compared with those obtained from collagenase-digested whole heart.

### RNA extraction, cDNA synthesis, and real-time RT-PCR

RNA was extracted from cultured/fractionated cells with the Aurum RNA Extraction Kit (Bio-Rad Laboratories, Hercules, CA, USA) or from heart tissue by Tri Reagent (MilliporeSigma, Burlington, MA, USA). cDNA was synthesized with a reverse-transcription system (Promega, Madison, WI, USA). Real-time RT-PCR was performed with an ABI-7500 System with Gene Expression Master Mix and specific Taqman primer/probe sets (Thermo Fisher Scientific, Waltham, MA, USA): *Ace* (Mm00802048_m1), *Agt* (Mm00599662_m1), *Col1a1* (Mm00801666_g1), *Col1a2* (Mm00483888_m1), *Col3a1* (Mm01254476_m1), *Ddr2* (Mm00445615_m1), *Fgf2* (Mm01285715_m1), *Igf1* (Mm00439560_m1), *Il6* (Mm00446190_m1), *Mapk14* (Mm00442498_m1), *Myh6* (Mm00440354_m1), *Myh7* (Mm00600555_m1), *Nppa* (Mm01255747_g1), *Pdgrfa* (Mm00440701_m1), *Pecam1* (Mm01242584_m1), *Ren1* (Mm02342887_mH), and *Tgfb1* (Mm01178820_m1). Data are expressed relative to the *Gapdh* housekeeping gene mRNA expression (Mm99999915_g1 primer/probes) with the cycle threshold (2^−Δ^*^Ct^*) method.

### Western blotting

Western blotting was performed on cell homogenates, as previously described (36, 37) using Cell Signaling Technology (New England BioLabs, Ipswich, MA, USA) antibodies for p38α (9228), phospho-p38 (9211), and phospho-HSP27 (2401). Horseradish peroxidase–conjugated anti-mouse and anti-rabbit secondary antibodies and enhanced chemiluminescence detection reagent were from GE Healthcare Life Sciences (Little Chalfont St. Giles, United Kingdom). mAb β-actin (ab8226) from Abcam (Cambridge, United Kingdom) was used as a loading control. The p38 inhibitor SB203580 (Merck, Kenilworth, NJ, USA) was used to evaluate the importance of p38α in mediating DAMP-induced changes in hypertrophic gene expression.

### Isoproterenol infusion

Alzet 1002 mini-osmotic pumps (Alzet, Cupertino, CA, USA) were implanted s.c. in isoflurane-anesthetized mice (control and Fb-p38α KO) as previously described by Ainscough *et al.* (37), and saline or ISO (30 mg/kg/d) was infused for 14 d. Pumps were removed under isoflurane anesthesia before recovery, and analysis of cardiac function occurred 1 wk later. Group sizes were the following: control-saline (*n* = 11), control-ISO (*n* = 9), Fb-p38α KO-saline (*n* = 8), and Fb-p38α KO-ISO (*n* = 11).

### Measurement of cardiac function and cardiac weight index

Physiological measurements of cardiac function were obtained at the end of the experimental period by Millar conductance pressure–volume (PV) catheter analysis as previously described by Frentzou *et al.* (38). Briefly, mice were anesthetized with isoflurane, and body temperature was maintained with a heating pad before inserting a 1.4 F miniature PV catheter (PVR-1045/SPR-839 Millar; AdInstruments, Bella Vista, NSW, Australia) into the left ventricle *via* the right carotid artery and ascending aorta. Data were collected *via* an MPVS-300 PV system (Millar; ADInstruments), and PV loop analysis was performed with Chart 8 Pro software (AdInstruments). Seven animals (18%) did not survive the PV procedure, resulting in final group sizes of control-saline (*n* = 9), control-ISO (*n* = 8), Fb-p38α KO-saline (*n* = 7), and Fb-p38α KO-ISO (*n* = 8). The investigator performing the PV measurements was blinded to the genotype of the animals.

Hearts were subsequently excised, cleaned, atria removed, and ventricles were weighed. Tibiae were collected, cleaned, and measured. Cardiac weight index was calculated as the ratio of ventricular weight to tibia length. Ventricles were snap frozen and were stored at −80°C for further analysis.

### Histology

Cryosections of ventricular tissue (8 μm thickness) were mounted on poly-l-lysine–coated slides and fixed with 4% paraformaldehyde for 20 min. Sections were incubated with rhodamine-labeled wheat germ agglutinin (WGA, 1:1000; Vector Laboratories, Burlingame, CA, USA) for 2 h, washed with PBS, then mounted with VectaShield containing DAPI (Vector Laboratories). Confocal images were captured with a Zeiss Axio Observer Z1m SP microscope (LSM700) with ×40 objective and Zeiss Zen 2.1 SP1 software (Carl Zeiss, Oberkochen, Germany).

Histological measurements were performed in a blinded fashion by 2 independent observers. Cardiomyocyte cross-sectional areas (37), and areas of interstitial fibrosis (37, 39) were quantified using Fiji–ImageJ software (National Institutes of Health, Bethesda, MD, USA), with at least 10 fields of view analyzed per animal, and 7–8 animals studied per group.

### MicroRNA RT-PCR array

cDNA was synthesized from 4 cardiac RNA samples for each of 3 groups (control-saline, control-ISO, and Fb-p38α KO-ISO) with the miScript II Reverse Transcription Kit (Qiagen, Hilden, Germany) before performing a miScript microRNA (miRNA) PCR array (MIMM-113ZA; Qiagen) with the ABI-7500 real-time PCR system. This SYBR-green–based array enabled expression levels of 84 cardiovascular-related miRNAs to be analyzed. Data are expressed relative to the geometric mean of the 6 normalization controls included on the array (SNORD61, SNORD68, SNORD72, SNORD95, SNORD96A, and RNU6-2) by the 2^−Δ^*^Ct^* method.

### Preparation of cardiac damage-associated molecular patterns

Murine hearts were excised, cleaned, and subjected to freeze/thaw and homogenization in 2 ml PBS per heart to disrupt tissue and cellular structure. The resultant homogenate was centrifuged to remove debris, and the supernatant was filter-sterilized before portioning into aliquots for long-term storage at −80°C. Those cardiac damage-associated molecular pattern (DAMP) preparations were used at a 1:10 dilution for cell culture studies. Several separate DAMP preparations were used throughout the study; all of which gave similar results.

### Statistical analysis

Statistical analyses were performed with Prism 6 software (GraphPad Software, La Jolla, CA, USA). All data are expressed as means ± sem, where *n* represents the number of separate animals investigated or the number of separate hearts from which cells were isolated. Data were analyzed by Student’s *t* test or 1-way ANOVA with the Sĭdák *post hoc* test, as appropriate. A value of *P* < 0.05 was considered statistically significant.

## RESULTS

### Generation of mice with inducible, fibroblast-specific deletion of p38α

To investigate the role of cardiac fibroblast p38α in hypertrophic cardiac remodeling, we generated an inducible, fibroblast-specific p38α KO mouse line, which involved crossing mice expressing fibroblast-specific, tamoxifen-inducible Cre recombinase [Col1a2-Cre-ER(T)] (29, 30) with mice expressing a modified p38α gene, *Mapk14*, with exons 2–3 (coding for the ATP-binding site of the kinase domain) flanked by loxP sites (31) (**Fig. 1*A*** and Supplemental Fig. 1). Tamoxifen injection of mice at 3 wk old induced Cre activity and the resultant Cre-lox–directed deletion of *Mapk14* exons 2–3 (Fig. 1*A*). Deletion was confirmed initially by PCR genotyping of ear notches (*i.e.*, dermal fibroblasts) (Fig. 1*B*) and confirmed in cell cultures. Primary cultures of cardiac fibroblasts from hearts of Fb-p38α KO mice had a 50% reduction of *Mapk14* mRNA (Fig. 1*C*) and p38α protein (Fig. 1*D*, *E*) compared with cells from control mice. Knockdown in freshly isolated cardiac cells was investigated by digesting hearts with collagenase before separating nonmyocytes into 2 distinct cell types with a MACS technique. Endothelial cells (*Pecam1*^+^ cells) were separated into fraction 1, along with leukocytes, whereas fibroblasts (*Ddr2*, *Pdgfra*, *Col1a1*, and *Col1a2*^+^) were separated into fraction 2 (Fig. 1*F*). Evaluation of relative *Gapdh* mRNA expression in the 2 fractions indicated that ∼64% of nonmyocytes were present in fraction 1 and 32% in fraction 2, in agreement with recent comprehensive studies by Pinto *et al.* (18) on the cellular composition of the murine heart. A 65% reduction in *Mapk14* mRNA levels was observed in the fibroblast-enriched fraction 2 from Fb-p38α KO mice compared with control mice (Fig. 1*G*). No reduction in *Mapk14* mRNA levels was evident in the endothelial cell/leukocyte-enriched fraction 1 (Fig. 1*G*), confirming the fibroblast-specific nature of the deletion. The extent of p38α depletion that we observed in isolated fibroblasts (65%) was similar to that reported by Lal *et al.* (32) in previous studies with the Col1a2-Cre-ER(T) approach.

### Fibroblast-specific p38α KO protects against catecholamine-induced cardiac hypertrophy *in vivo*

**Figure 2:**
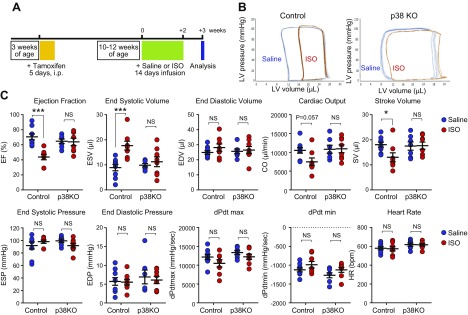
Effect of fibroblast-specific p38α KO on ISO-induced cardiac dysfunction. *A*) Timeline for chronic β-adrenergic receptor activation model of cardiac hypertrophy. *B*) Individual representative PV loops obtained from control and fibroblast-specific p38α KO mice following infusion with either saline (control, blue) or ISO (red). *C*) PV conductance catheter data. Individual data and means ± sem are shown. Group sizes: control saline (*n* = 9), control ISO (*n* = 8), KO saline (*n* = 7), and KO ISO (*n* = 8). EDV, end diastolic volume; CO, cardiac output; SV, stroke volume. ANOVA with Sĭdák *post hoc* test: *P < 0.05, ***P < 0.001. NS, not significant.

The effect of fibroblast-specific p38α KO was investigated in a model of cardiac hypertrophy induced by chronic β-adrenergic stimulation. Control or Fb-p38α KO mice were injected with tamoxifen at 3 wk old, and then, at 10–12 wk old, they were implanted with osmotic minipumps delivering saline or ISO (30 mg/kg/d) for 14 d (**Fig. 2*A***). Pumps were removed, and the animals were left for 1 additional week before analyzing cardiac function by PV catheter recordings (Fig. 2*B*). In control mice, characteristic ISO-induced cardiac dysfunction and dilatation were observed, as measured by several hemodynamic indices, including reduced ejection fraction (EF; control = 70.6 ± 4.0, ISO = 43.7 ± 3.6%; *P* < 0.001), reduced stroke volume (control = 18.0 ± 1.0, ISO = 13.0 ± 1.8 μl; *P* < 0.05), reduced cardiac output (control = 10,509 ± 744, ISO = 7476 ± 1028 μl/min; *P* = 0.06), and increased end systolic volume (ESV; control = 8.9 ± 1.3, ISO = 17.7 ± 1.8 μl; *P* < 0.001) (Fig. 2*C*). Fb-p38α KO mice exhibited remarkable protection against ISO-induced cardiac dysfunction. In Fb-p38α KO mice, ISO treatment did not significantly affect EF (ISO = 63.9 ± 4.8%), stroke volume (ISO = 17.6 ± 1.4 μl), cardiac output (ISO = 10,940 ± 1018 μl/min), or ESV (ISO = 11.2 ± 2.0 μl) (Fig. 2*C*).

**Figure 3:**
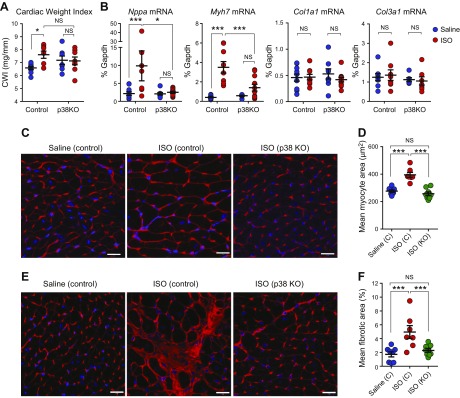
Effect of fibroblast-specific p38α KO on isoproterenol-induced cardiac hypertrophy. Control or Fb-p38α KO mice were injected with tamoxifen and mini-osmotic pumps implanted for delivery of saline or ISO as in Fig. 2*A*. Pumps were removed and heart tissue collected 1 wk later. *A*) Ventricular weight/tibia length ratio (cardiac weight index) from animals used in PV analysis. Group sizes: control saline (*n* = 9), control ISO (*n* = 8), KO saline (*n* = 7), and KO ISO (*n* = 8). *B*) Real-time RT-PCR analysis of cardiac mRNA levels for cardiomyocyte hypertrophy markers atrial natriuretic factor (*Nppa*) and β-myosin heavy chain (*Myh7*) and fibrosis markers *Col1a1* and *Col3a1*. Group sizes: control saline (*n* = 11), control ISO (*n* = 9), KO saline (*n* = 8), and KO ISO (*n* = 11). *C*) Representative images of WGA-labeled heart sections used to determine myocyte cross-sectional area. *D*) Mean cardiomyocyte size (cross-sectional area) determined from WGA-stained images. Group sizes: control saline (*n* = 8), control ISO (*n* = 7), and KO ISO (*n* = 8). *E*) Representative images of WGA-labeled heart sections used to determine areas of interstitial fibrosis. *F*) Mean interstitial fibrotic area determined from WGA-stained images. Group sizes: control saline (*n* = 8), control ISO (*n* = 7), and KO ISO (*n* = 8). NS, not significant. Scale bars, 20 μm. Individual data and means ± sem are shown. *P < 0.05, **P < 0.01, ***P < 0.001 (ANOVA with Šidák *post hoc* test).

Cardiac hypertrophy was investigated by measuring cardiac weight index (ventricular weight/tibia length ratio), expression of hypertrophy-associated fetal cardiomyocyte genes and cardiomyocyte cross-sectional area (**Fig. 3**). In control mice, ISO induced significant increases of 9% in cardiac weight index (Fig. 3*A*), 4.6-fold in atrial natriuretic factor (*Nppa*) mRNA expression, 8.6-fold in β-MHC (*Myh7*) mRNA expression (Fig. 3*B*) and 43% in cardiomyocyte cross-sectional area (Fig. 3*C*, *D*). Strikingly, Fb-p38α KO mice showed very little evidence of ISO-induced cardiac hypertrophy measured by any of these methods (Fig. 3*A–D*). ISO infusion also promoted significant interstitial fibrosis in control mice, but not in Fb-p38α KO mice, as evidenced by WGA staining (Fig. 3*E*, *F*). No differences were evident in collagen mRNA expression (*Col1a1* or *Col3a1*) at the end of the experiment (Fig. 3*B*), suggesting much of the active remodeling had been resolved by this 3-wk point.

**Figure 4:**
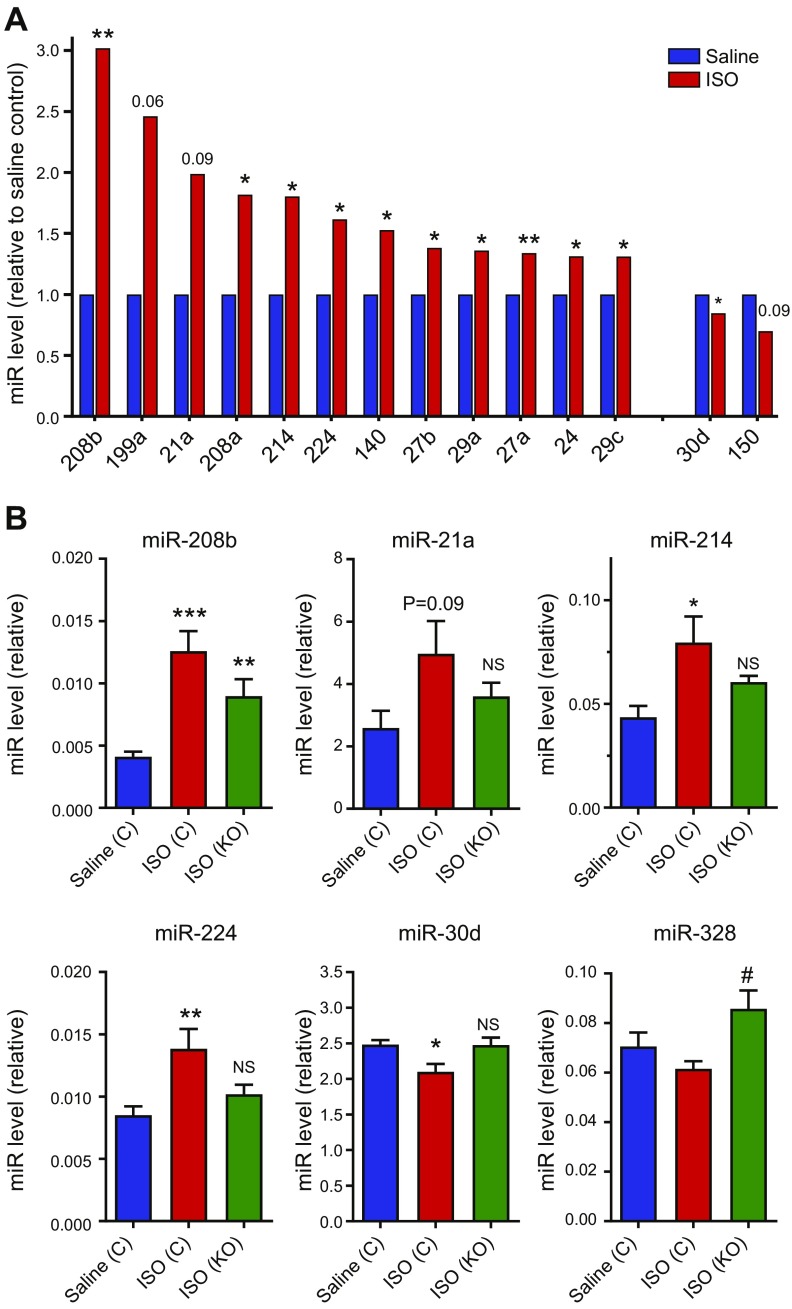
Effect of fibroblast-specific p38α KO on isoproterenol-induced miRNA expression. Control or Fb-p38α KO mice were injected with tamoxifen and infused with saline or ISO as described in Figs. 2*A* and 3 legend. Heart tissue was collected 1 wk after removal of miniosmotic pumps and expression levels of 84 cardiovascular miRNAs determined using a real-time RT-PCR array. Group sizes: *n* = 4. See Supplemental Table 1 for full data set. *A*) miRNAs (miR) increased or decreased following ISO infusion. *P < 0.05, **P < 0.01 for effect of ISO (unpaired Student’s *t* test). *B*) miRNAs modulated by fibroblast-specific p38α KO. *P < 0.05, **P < 0.01, ***P < 0.001 (ANOVA with Šidák *post hoc* test). NS, not significant, compared with control saline group. ^#^*P* < 0.05, compared with control ISO group. Data expressed relative to array-normalization controls.

A focused miRNA array was employed to investigate the effect of ISO on selected cardiovascular miRNAs and the influence of fibroblast-specific p38α KO (**Fig. 4** and **Table 1**). RNA samples prepared from hearts of saline-infused control mice were compared with those of ISO-infused control mice and ISO-infused Fb-p38α KO mice (4 hearts/group), and expression levels of 84 cardiovascular miRNAs were evaluated (see Supplemental Table 1 for full data set). The 10 most highly expressed miRNAs in control hearts from saline-infused animals included miR-1a, miR-126a, miR-24, and multiple members of the miR-23, miR-26 and miR-30 families (Table 1). Of the 84 miRNAs studied, 12 were reproducibly increased in hypertrophic hearts from ISO-infused mice compared with hearts from saline-infused mice (miR-21a, -24, -27a/b, -29a/c, -140, -199a, -208a/b, -214, and -224), and 2 miRNAs were decreased in ISO hearts compared with saline hearts (miR-30d and -150) (Fig. 4*A*). Fibroblast-specific p38α KO opposed the effects of ISO on some of those miRNAs, namely, miR-208b, -21a, -214, -224, and -30d (Fig. 4*B*). P38α KO also induced miR-328 expression, although it was not modulated by ISO, suggesting negative regulation of that miRNA by fibroblast p38α (Fig. 4*B*).

**TABLE 1:** Expression of miRNAs in control hearts

miRNA	Expression (2^−Δ^*^Ct^*)
1a	75.4
126a	18.1
30c	11.5
23a	8.2
26a	5.6
26b	5.6
30a	4.7
23b	4.6
24	4.6
30e	4.1
125b	3.9
195a	3.3
451a	3.1
16	2.8
27a	2.7
let-7f	2.7
378a	2.7
21a	2.6
30d	2.5
125a	2.1
29c	2.1
29a	2.0

Control (Cre-negative) mice were injected with tamoxifen and infused with saline, as described in Fig. 2*A*. Heart tissue was collected 1 wk after removal of miniosmotic pumps, and expression levels of 84 cardiovascular miRNAs were determined using a real-time RT-PCR array. Group sizes: *n* = 4. See Supplemental Table 1 for full data set. Table lists the 22 most highly expressed miRNAs in control hearts from saline-infused mice. Data are mean expression levels (2^−Δ^*^Ct^*) relative to array-normalization controls.

Taken together, these *in vivo* data demonstrate that fibroblast-specific p38α KO confers cardioprotective benefits by attenuating ISO-induced cardiac hypertrophy and dysfunction. Given that cardiac fibroblasts have a critical role in stimulating cardiomyocyte hypertrophy through paracrine secretion of hypertrophic growth factors, we hypothesized that cardiac fibroblast p38α was important for secretion of such factors and could, thereby, stimulate cardiomyocyte hypertrophy.

### Cardiac fibroblast p38α is required for damage-induced secretion of the cardiomyocyte hypertrophy-inducing factor IL-6

**Figure 5:**
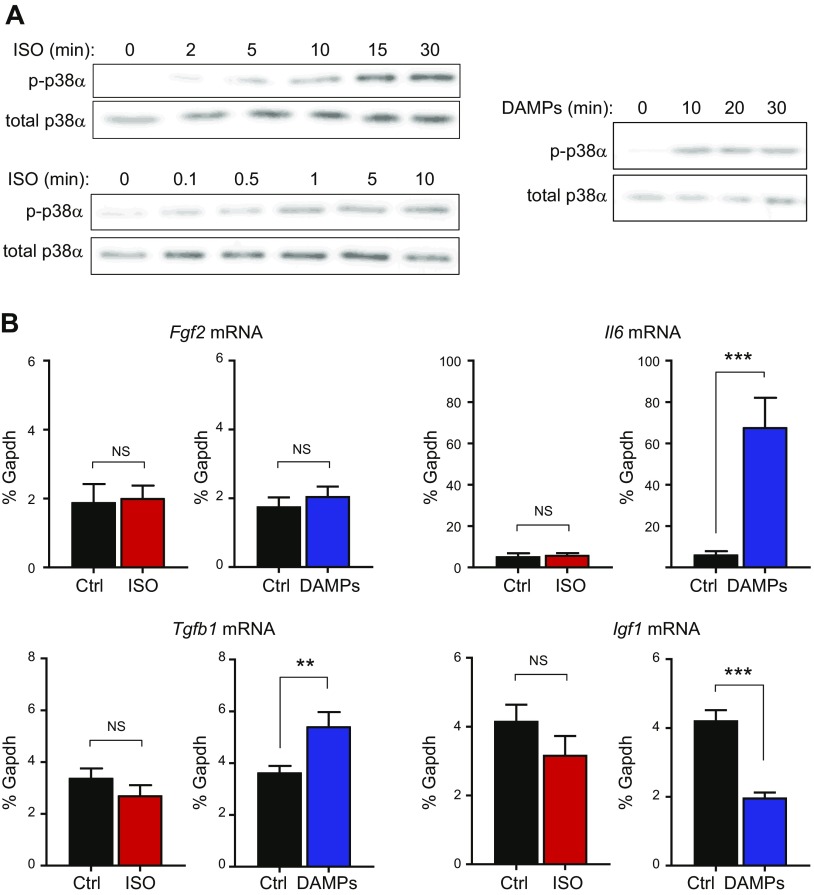
Effects of isoproterenol and cardiac DAMPs on p38α activation and expression of hypertrophy-inducing factors in cultured cardiac fibroblasts. *A*) Western blotting of phosphorylated (activated) p38α and total p38α expression showing time of response to 5 μM ISO, concentration response to 0.1–10 μM ISO after 15 min, and time of response to cardiac DAMPs. Blots are representative of 3 separate experiments. *B*) Real-time RT-PCR data showing effect of ISO (5 μM, 6 h, *n* = 8) or cardiac DAMPs (6 h, *n* = 12) on mRNA expression of *Fgf2*, *Il6*, *Tgfb1* and *Igf1*. Data expressed as percentage *Gapdh* mRNA levels. **P < 0.01, ***P < 0.001. NS, not significant (paired-ratio Student’s *t* test).

We first investigated whether ISO could directly activate p38 MAPK in cultured cardiac fibroblasts, and then, whether it could induce specific myocyte hypertrophy-inducing factors (FGF-2, IGF-1, TGF-β1, and IL-6) in a p38α-dependent manner. Although ISO directly activated fibroblast p38α (**Fig. 5*A***), it was unable to stimulate expression of any of the hypertrophy-inducing genes tested (Fig. 5*B*).

We next investigated whether ISO could be modulating fibroblast function indirectly by inducing cardiac damage, resulting in release of DAMPs and fibroblast activation (20). To mimic that *in vitro*, we prepared cardiac DAMPs by freeze-thawing and homogenizing mouse heart tissue. That cardiac DAMP preparation activated p38α when added to cultured cardiac fibroblasts (Fig. 5*A*). Cardiac DAMPs did not modulate *Fgf2* mRNA levels but strongly stimulated *Il6* mRNA expression by >10-fold and increased *Tgfb1* mRNA levels by 50% after 6 h (Fig. 5*B*). The DAMP preparation had the opposite effect on *Igf1* mRNA levels, decreasing them by 50% (Fig. 5*B*).

The cardiac DAMP preparation represented a heterogeneous mixture of cellular proteins, nucleic acids, and biochemicals, mimicking the DAMPs released after cardiac damage that induces an innate immune response (20, 40). We investigated which specific components of the cardiac DAMP preparation were responsible for activating p38α and IL-6 expression in cardiac fibroblasts (Supplemental Fig. 2). Those experiments involved *1*) testing its efficacy in cardiac fibroblasts that lacked the IL-1 receptor IL-1R1 because IL1α has been shown to be a potential DAMP in this setting (41), and *2*) investigating whether inhibition of the TLR4 would inhibit the effects of DAMPs because that pattern recognition receptor has been shown to mediate the effects of several DAMPs on cardiac fibroblasts (20). Our studies revealed that the effects of the cardiac DAMP preparation on cardiac fibroblasts occurred independent of IL-1R1 or TLR4 (Supplemental Fig. 2).

**Figure 6:**
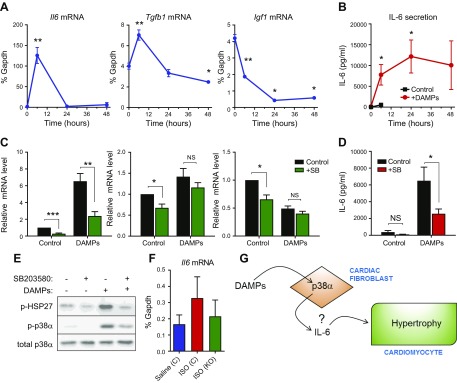
Role of p38α in DAMPs-modulated expression of hypertrophy-inducing genes in cardiac fibroblasts. *A*) Real-time RT-PCR showing time of effect of DAMPs on mRNA expression of *Il6*, *Tgfb1*, and *Igf1*. *P < 0.05, **P < 0.01 (*n* = 3). *B*) ELISA showing time of IL-6 secretion from cardiac fibroblasts stimulated with cardiac DAMPs. Red filled circles represent DAMP-stimulated IL-6 secretion, and black-filled squares represent basal secretion without addition of DAMPs (measured up to 6 h only). *P < 0.05 compared with time 0 (*n* = 3). *C*) Real-time RT-PCR data showing effect of 10 μM SB203580 or DMSO vehicle control on DAMP-induced expression of hypertrophy-inducing genes after 6 h (*n* = 9). *P < 0.05, **P < 0.01, ***P < 0.001 (ANOVA with Šidák *post hoc* test). NS, not significant. Data normalized to *Gapdh* mRNA levels and expressed relative to the control. *D*) ELISA showing effect of 10 μM SB203580 or DMSO vehicle control on DAMP-induced IL-6 secretion after 6 h (*n* = 9). ANOVA with Sĭdák *post hoc* test. *P < 0.05; NS, not significant. *E*) Western blot showing DAMP-induced phosphorylation (p-) of HSP27 and p38α after 20 min and inhibition by 10 μM SB203580. Total p38α expression was included as the loading control. Blots are representative of 3 separate experiments. *F*) Control or Fb-p38α KO mice were injected with tamoxifen, and miniosmotic pumps were implanted for delivery of saline or ISO as in Fig. 2*A*. Pumps were removed, and heart tissue was collected 1 wk later. Bar chart shows real-time RT-PCR analysis of cardiac *Il6* mRNA levels (means ± sem). Group sizes: control saline (*n* = 11), control ISO (*n* = 9), and KO ISO (*n* = 11). ANOVA with Sĭdák *post hoc* test: not significant. *G*) Schematic depicting role of fibroblast p38α in modulating cardiomyocyte hypertrophy.

Further investigation into the timing of the effects of cardiac DAMPs on gene expression revealed that the increase in *Il6* and *Tgfb1* mRNA expression returned to basal levels within 24 h, whereas the reduction in *Igf1* levels was maintained for at least 48 h (**Fig. 6*A***). Despite the relatively transient nature of *Il6* mRNA expression, ELISA analysis of the conditioned medium confirmed a sizeable elevation in IL-6 protein secretion in response to cardiac DAMPs, with peak levels of >10 ng/ml maintained for 24–48 h after the initial stimulus (Fig. 6*B*). The p38 inhibitor SB203580 was used to evaluate the importance of p38α in mediating DAMP-induced changes in hypertrophic gene expression. SB203580 prevented p38α downstream signaling, as expected (Fig. 6*E*), and significantly reduced DAMP-induced *Il6* mRNA expression and protein secretion (Fig. 6*C*, *D*). In contrast, the p38 inhibitor had no effect on DAMP-induced expression of *Tgfb1* or *Igf1* mRNA (Fig. 6*C*). Together these data indicate that cardiac DAMPs can stimulate IL-6 transcription and protein secretion in a p38α-dependent manner; a mechanism that may underlie our *in vivo* observations on cardiac hypertrophy.

To evaluate whether IL-6 was up-regulated by chronic ISO infusion *in vivo* and modulated by fibroblast-specific p38α KO, we measured *Il6* mRNA levels in whole-heart samples at the end of the experimental protocol. Those data (Fig. 6*F*) revealed a trend toward increased *Il6* mRNA expression in hearts from ISO-infused control mice, compared with those with saline infusion, and a trend toward decreased *Il6* mRNA in hearts from ISO-infused, fibroblast-specific p38α KO mice compared with ISO-infused control mice.

To determine whether fibroblast p38α was important for expression of the renin–angiotensin system (RAS) genes in fibroblasts and, thereby, potentially contributing to our *in vivo* findings by inhibiting local RAS activation, we investigated the effect of the p38 inhibitor SB203580 on RAS gene expression in cultured mouse cardiac fibroblasts (Supplemental Fig. 3*A*). Neither angiotensinogen (*Agt*) nor angiotensin-converting enzyme (*Ace*) mRNA levels were affected by 6 h of exposure to SB203580, conditions that reduced *Il6* mRNA levels by >50%. Moreover, the cardiac DAMP preparation had the opposite effect on *Agt* and *Ace* expression, compared with *Il6* expression, reducing their mRNA levels by 40–50% (Supplemental Fig. 3*B*). Renin (*Ren1*) mRNA was not detectable in any of these samples.

## DISCUSSION

Our study demonstrated that fibroblast-specific KO of p38α negates the deleterious effects of chronic β-adrenergic receptor stimulation on cardiac hypertrophy and function. Specifically, our *in vivo* experiments revealed that fibroblast-specific p38α KO prevented the ability of chronic ISO infusion to reduce EF, increase ESV, increase the ratio of ventricular weight/tibia length, increase cardiomyocyte area, increase fibrosis, up-regulate myocyte hypertrophy markers (atrial natriuretic factor, β-MHC), up-regulate prohypertrophic miRNAs, and down-regulate antihypertrophic miRNAs. Our supporting *in vitro* experiments indicated a key role for p38α in mediating DAMP-induced secretion of the cardiomyocyte hypertrophy-inducing factor IL-6 from cardiac fibroblasts. Our study, therefore, suggests that cardiac fibroblast p38α is important for regulating cardiomyocyte hypertrophy through paracrine fibroblast-to-myocyte signaling involving IL-6 secretion (Fig. 6*G*).

Despite nearly 2 decades of study, the precise, individual roles of p38α and p38β in modulating cardiomyocyte hypertrophy remain unclear, with seemingly contradictory outcomes reported (2, 3, 10). Results from *in vitro*, ectopic, overexpression studies of individual p38 subtypes in cardiomyocytes are at odds with cardiomyocyte-targeted *in vivo* genetic inhibition studies, yet pharmacologic p38α inhibition appears effective at reducing cardiac hypertrophy. Our data offer a potential unifying explanation by uncovering a role for cardiac fibroblast p38α in modulating cardiomyocyte hypertrophy *in vivo*.

Many stimuli that cause LV hypertrophy (*e.g.*, catecholamines, Ang II, and pressure overload) are direct inducers of cardiomyocyte cellular hypertrophy *in vitro*, and that has been assumed to underlie their hypertrophic action on the heart. However, that concept has been challenged by several recent studies showing that fibroblasts act as primary integrators of hypertrophic stimuli in the intact heart (22, 23). A number of paracrine-signaling molecules have been identified through which fibroblasts can modulate cardiomyocyte hypertrophy, including FGF2, IGF-1, and TGF-β. Additionally, we hypothesized that IL-6 may act in this manner because that cytokine is up-regulated in mouse hearts after 1–2 wk of ISO infusion (42, 43), is actively secreted from cardiac fibroblasts in response to β-adrenergic receptor stimulation or Ang II (19), and is able to directly induce cardiomyocyte hypertrophy (44, 45), and IL-6 KO mice are protected against LV hypertrophy in response to noradrenaline, Ang II, or pressure overload (44, 46, 47). Moreover, cardiomyocyte hypertrophy induced *in vitro* by either Ang II or phenylephrine is impaired in myocytes isolated from IL-6 KO mice (44), suggesting that autocrine IL-6 secretion is necessary for induction of myocyte hypertrophy by those stimuli. A diverse range of stimuli have been reported to induce IL-6 expression in cardiac fibroblasts in a p38-dependent manner, including IL-1, IL-33, TNF-α, Ang II, adenosine, and α/β-adrenergic receptor agonists (1, 48, 49). Thus, the p38 pathway appears to have a fundamental role in stimulating IL-6 secretion from cardiac fibroblasts in response to a variety of molecules up-regulated during cardiac injury and remodeling. In agreement with our hypothesis, a key paracrine role for fibroblast-derived IL-6 has recently been found to account for the cardiac hypertrophy observed in a mouse overexpressing fibroblast/smooth-muscle cell–targeted sphingosine 1 phosphate receptor-1 (50). Those previous studies, together with our current *in vitro* mechanistic data, suggest a scenario whereby cardiac fibroblast p38α stimulates cardiomyocyte hypertrophy by sensing damage to the heart and inducing IL-6 paracrine crosstalk (Fig. 6*G*). However, the role of fibroblast p38α in regulating cardiomyocyte hypertrophy is likely to be more complex than simply altering the secretion of a single paracrine hypertrophic factor. Further studies will, therefore, be required to unequivocally define the importance of cardiac fibroblast-derived IL-6 in this setting.

The miRNA microarray identified several highly expressed miRNAs in the murine heart tissue of controls, with miR-1, -126a, and -30c being the most abundant. Of the 84 miRNAs studied, 12 were reproducibly increased, and 2 were decreased in hypertrophic hearts from ISO-infused mice compared with hearts from saline-infused mice. Many of these miRNAs have been shown to be modulated similarly in other reports on mouse and rat models of cardiac hypertrophy (51–55). We identified 5 miRNAs that were significantly regulated by ISO in control mice but not in Fb-p38α KO mice (miR-21a, -214, -224, -208b, and -30d), suggesting they are important for the cardioprotective effect of fibroblast-specific p38α KO. Both miR-21 and miR-214 are highly expressed by cardiac fibroblasts and have important roles in myocardial remodeling (56–59). Notably, both those miRNAs positively regulate IL-6 expression in macrophages and fibroblast-like ligamentum flavum cells (60, 61) and may, therefore, have similar roles in cardiac fibroblasts, contributing to the paracrine IL-6 hypertrophic effect we describe. Recent studies have shown that modulation of miR-21 in cardiac fibroblasts *in vivo* positively regulates paracrine hypertrophic signaling induced by pressure overload (56). Much less well studied is miR-224 in the heart, but it is also expressed by cardiac fibroblasts, and its levels are up-regulated by Ang II (62). Potential target genes for miR-224 include several that regulate cardiac fibroblast proliferation and cardiac fibrosis (62). MiR-208b is cardiomyocyte specific because it is located within an intron of the β-MHC (*Myh7*) gene (63); hence, its increase was expected, given the increase in *Myh7* mRNA expression observed in ISO hearts. Finally, miR-30d, which we found was reduced in control hypertrophic hearts but not fibroblast-specific p38α KO hearts, has previously been shown to be reduced with pressure overload–induced cardiac hypertrophy (53). Interestingly, several other members of the miR-30 family are also known to regulate cardiac hypertrophy and fibrosis (64, 65). We identified just 1 miRNA (miR-328) that was elevated in ISO-infused p38 KO hearts, compared with ISO-infused control hearts, suggesting negative regulation by fibroblast p38α. Indeed, miR-328 has been shown to be negatively regulated by p38 in human osteosarcoma cells (66). Furthermore, miR-328 was recently reported to regulate myocardial fibrosis *via* effects on cardiac fibroblast function (67).

A marked elevation of cardiac *Il6* mRNA levels has previously been reported LV samples from ISO-infused mice after 1–2 wk (42, 43). We observed a trend toward increased *Il6* mRNA levels in hearts from ISO-treated control mice after 3 wk, which was not apparent in hearts from ISO-treated fibroblast-specific p38 KO mice. However, *Il6* mRNA levels were low and the differences were not statistically significant, which may be explained by samples being derived from whole heart [in which fibroblasts account for <20% of cells (18)] and at the end of the experimental protocol (*i.e.*, 1 wk after cessation of ISO treatment); at which time, the effects of ISO on *Il6* mRNA expression were likely to be less pronounced (42).

Although we did not identify the precise bioactive molecule(s) in the cardiac DAMP preparation responsible for p38α activation and IL-6 expression in cardiac fibroblasts, we did establish that those effects occurred independent of the IL-1R1 or TLR4 receptors. There are several other candidate molecules and receptors that could fulfill that role (20, 40), including IL-18 (an IL-1 family cytokine that acts *via* a different receptor), which has recently been shown to be up-regulated in response to ISO treatment in mice, and its release from damaged cardiomyocytes triggered inflammation and pathologic cardiac remodeling (68). We also cannot rule out that combinations of molecules in the DAMP preparation were driving IL-6 secretion, rather than a single, specific molecule, especially given the diversity of molecules up-regulated in the remodeled heart that are known to induce IL-6 secretion from fibroblasts in a p38-dependent manner (1, 48, 49). Hence, more in-depth analyses, beyond the scope of the current study, would be required to identify the precise molecular components of the cardiac DAMP preparation that trigger p38α activation and IL-6 production in cardiac fibroblasts.

The relationship between the adrenergic system and the RAS in the setting of cardiac remodeling is complex, with several points of interaction (69). Rat cardiac fibroblasts are reported to express angiotensinogen, renin, and angiotensin 1–converting enzyme, thereby contributing to both a local myocardial RAS and an intracellular RAS (70). In contrast to that report, we did not detect *Ren1* mRNA in murine cardiac fibroblasts; whether that is a species-specific difference requires further study. Under basal conditions, *Agt* and *Ace* mRNA were not regulated by p38α, which agrees with a previous study in rat cardiac fibroblasts (71). Cardiac DAMPs reduced both *Agt* and *Ace* mRNA expression, contrary to the effects on *Il6* mRNA expression. Together, these data appear to rule out a mechanism in which fibroblast-specific p38α KO directly reduced myocardial RAS activation *in vivo*.

Several reports have emerged recently that support a critical role for cardiac fibroblast p38α in modulating cardiac remodeling (27, 72, 73). For example, a nonbiased transcriptomic approach identified cardiac fibroblast ATF3 as being cardioprotective in heart-failure models; and fibroblast p38 was identified as the downstream molecule responsible for profibrotic and hypertrophic effects in fibroblast-specific ATF3 KO mice [Col1a2-Cre-ER(T) model] (73). In a second study, global knockdown of MK5 (a p38 substrate localized to cardiac fibroblasts but not to myocytes) was associated with the attenuation of both hypertrophy and cardiac dysfunction in response to chronic pressure overload (72). Recently, it was reported that inducible fibroblast-selective knockdown of p38α (in *Tcf21*- and *Postn*-directed Cre KO mouse models) could reduce myofibroblast differentiation and fibrosis after ischemic injury or chronic neurohumoral stimulation (27). Although focused on fibrosis, that study also noted that fibroblast-selective p38α KO mice had reduced cardiac hypertrophy induced by Ang II and phenylephrine infusion (27). Our study defines a clear hypertrophic role for cardiac fibroblast p38α and identifies IL-6 as a possible p38α-induced paracrine factor capable of stimulating cardiomyocyte hypertrophy in this setting. Thus, strong evidence is accumulating that p38α in cardiac fibroblasts acts as a central mediator of cardiac hypertrophy and fibrosis in a variety of pathologic scenarios, making it an attractive target for therapeutic intervention.

In summary, our study reveals an important role for p38α, specifically in cardiac fibroblasts, in stimulating cardiac hypertrophy after chronic β-adrenergic stimulation, potentially *via* an IL-6–dependent mechanism. These findings help to explain the disparity between the effects of pharmacologic p38 inhibitors and cardiomyocyte-specific KO/inhibition models for inhibiting cardiac hypertrophy *in vivo*. They also further our understanding of the key role that cardiac fibroblasts have in regulating cardiac hypertrophy and remodeling through paracrine signaling, and our findings identify the cardiac fibroblast p38α/IL-6 axis as a potential therapeutic target in this setting.

## Supplementary Material

This article includes supplemental data. Please visit *http://www.fasebj.org* to obtain this information.

## Abbreviations

Ang II: angiotensin II
DAMP: damage-associated molecular pattern
EF: ejection fraction
ESV: end systolic volume
Fb-p38α KO: fibroblast-specific p38α knockout
FGF2: fibroblast growth factor 2
IL-1R1: IL-1 receptor
ISO: isoproterenol
KO: knockout
LV: left ventricular
MACS: magnetic antibody cell separation
MHC: myosin heavy chain
miRNA: microRNA
PV: pressure–volume
RAS: renin–angiotensin system
WGA: wheat germ agglutinin
