# Supplementary material for: Cardiac fibroblast-specific p38α MAP kinase promotes cardiac hypertrophy via a putative paracrine interleukin-6 signaling mechanism
Source: FASEB J. 2018 Mar 30;32(9):4941–54. doi: 10.1096/fj.201701455RR (PMC6629170; doi:10.1096/fj.201701455RR)
Supplement: Supplementary file 1 [file fj.201701455RR.sd1.docx]

**
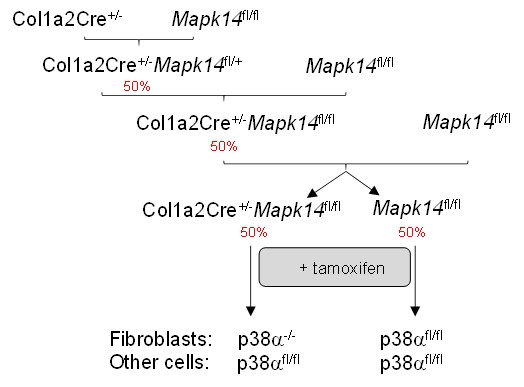
**

**Supplemental Figure 1.** Breeding strategy for generating Col1a2-Cre-ER(T) positive (experimental) and Col1a2-Cre-ER(T) negative (control) *Mapk14*^fl/fl^ mice. Percentages indicate predicted proportion of correct genotype.

**Supplemental Figure 2.** Effects of IL-1 receptor (IL-1R1) knockout or Toll-like receptor-4 (TLR4) inhibition on p38α activation and IL-6 expression in mouse cardiac fibroblasts. **(A)** Western blot of DAMPs-induced p38α phosphorylation after 20 min in cardiac fibroblasts from wild-type (WT) mice or global IL-1R1 knockout (KO) mice (n=3) showing no difference in responses. Note that in parallel experiments, it was confirmed that p38α activation by IL-1α was fully inhibited in cells from IL-1R1 KO mice, confirming effective IL-1R1 deletion (data not shown; Hemmings KE et al, manuscript in preparation). **(B)** Effect of DAMPs on *Il6* mRNA expression after 6 h in WT and IL-1R1 KO mice (n=3), showing no difference in responses. Note that in parallel experiments, *Il6* mRNA induction by IL-1α was fully inhibited in cells from IL-1R1 KO mice, confirming effective IL-1R1 deletion (data not shown; Hemmings KE et al, manuscript in preparation). ^NS^ not significant for effect of IL-1R1 KO. **(C)** Effect of TLR4 inhibitor TAK-242 (Merck; 2 μM) on *Il6* mRNA expression induced by cardiac DAMPs or 1 μg/ml LPS (TLR4 agonist) after 6 h (n=4). **P<0.01, ^NS^ not significant for effect of TAK-242.

**A.**

**B.**

**Supplemental Figure 3.** Effect of p38 inhibition and cardiac DAMPs on expression of components of the renin-angiotensin system in mouse cardiac fibroblasts. Real-time RT-PCR data showing effect of **(A)** p38 inhibitor SB203580 (10 μM) or **(B)** cardiac DAMPs on expression of *Agt* (angiotensinogen), *Ace* (angiotensin converting enzyme), *Ren1* (renin) and *Il6* (interleukin-6) genes 6 h after treatment. Data expressed as % *Gapdh* mRNA levels (n=4). ***P<0.001, **P<0.01, ^NS^ not significant.

**Supplemental Table 1.** Effect of fibroblast-specific p38α knockout on isoproterenol-induced microRNA expression - complete data set. See Fig. 4 legend for experimental details. A01-G12 = mean expression levels (2^-ΔCT^) of 84 cardiovascular microRNAs relative to normalization controls. H01-H02 = negative controls; H03-H08 = normalization controls; H09-H10 = reverse transcription positive controls; H11-H12 = PCR positive controls.
